# Amino Acid Mutation in Position 349 of Glycoprotein Affect the Pathogenicity of Rabies Virus

**DOI:** 10.3389/fmicb.2020.00481

**Published:** 2020-04-03

**Authors:** Jun Luo, Boyue Zhang, Yuting Wu, Xiaofeng Guo

**Affiliations:** College of Veterinary Medicine, South China Agricultural University, Guangzhou, China

**Keywords:** rabies virus, mutation, pathogenicity, glycoprotein, immunogenicity

## Abstract

Rabies, caused by rabies virus (RABV), is a zoonotic disease infecting mammals including humans. Studies have confirmed that glycoprotein (G) is most related to RABV pathogenicity. In the present study, to discover more amino acid sites related to viral pathogenicity, artificial mutants have been constructed in G of virulent strain GD-SH-01 backbone. Results showed that pathogenicity of GD-SH-01 significantly decreased when Gly_349_ was replaced by Glu_349_ through *in vivo* assays. Gly_349_→Glu_349_ of G did not significantly influence viral growth and spread in NA cells. Gly_349_→Glu_349_ of G increased the immunogenicity of GD-SH-01 in periphery and induced more expression of interferon alpha (IFN-α) in the brain in mice. It was observed that Gly_349_→Glu_349_ of G led to enhanced blood–brain barrier (BBB) permeability at day 5 postinfection. All together, these data revealed that Gly_349_→Glu_349_ of G mutation decreased RABV pathogenicity through enhanced immune response and increased BBB permeability. This study provides a new referenced site G349 that could attenuate pathogenicity of RABV.

## Introduction

Rabies is an ancient zoonotic disease affecting the central nervous system (CNS) and continues to be a worldwide health problem. In humans, once the rabies symptoms manifest, the mortality rate is almost 100%. The causative agent of rabies is the rabies virus (RABV), which causes fatal encephalitis in warm-blooded animals (Jackson, 2003). RABV, an unsegmented, negative-stranded RNA virus, belongs to the genus *Lyssavirus* of the family *Rhabdoviridae*. The RABV genome is ∼12 kb in size and comprises five genes, encoding nucleoprotein (N), phosphoprotein (P), matrix protein (M), glycoprotein (G), and the RNA-dependent RNA polymerase (L) (Schnell et al., 2010). Glycoprotein, the sole protein exposed on the surface of the virion, is the most important determinant of RABV pathogenicity and is the major protein to induce virus neutralizing antibody (VNA) (Dietzschold et al., 1983; Morimoto et al., 1999; Ito et al., 2001; Faber et al., 2002, 2004). Different RABV strains possess different virulence mainly based on the different sequences of G proteins. Amino acid residues 333, 194, 37, and 242/255/268 in G have been demonstrated to be strongly related to pathogenicity (Faber et al., 2005; Takayama-Ito et al., 2006a, b; Yamada et al., 2014b). However, mutations in the G protein that contribute to the pathogenicity are sometimes strain dependent (Takayama-Ito et al., 2004, 2006b). Therefore, it is likely that some amino acid residues correlated to virulence have still not been discovered and reported in the literature. RABV G is comprised of a signal peptide, an ectodomain, a transmembrane domain, and a cytoplasmic tail (Wunner et al., 1988; Kuzmina et al., 2013). The ectodomain and cytoplasmic of G have been linked to the pathogenicity and immune evasion of the virus (Coulon et al., 1998; Prehaud et al., 2010; Virojanapirom et al., 2012; Huang et al., 2017). In addition, four major and one minor antigenic sites of G were identified using monoclonal antibodies (Lafon et al., 1984; Benmansour et al., 1991). Residues 34–42 and 198–200 of G have been recognized antigenic site II (Prehaud et al., 1988). Previous studies suggest that site III is from amino acids 330 to 338 in G (Seif et al., 1985; Wunner et al., 1988). Amino acid arginine at position 333 of G, which locates in site III, is virulent for adult mice (Dietzschold et al., 1983; Tuffereau et al., 1989). Therefore, most of the antigenic sites described locate in ectodomain of glycoprotein.

A highly pathogenic GD-SH-01 strain, which caused pigs’ death in a farm in Southern China, was isolated from a rabid pig (Luo et al., 2013). The analysis of whole-genome phylogeny and the comparison of nucleotide acid sequences suggested that the GD-SH-01 strain was closely associated with clade I of China, and it was found to be more pathogenic than challenge virus standard 24 (CVS-24) based on the pathogenicity index comparison (Luo et al., 2013). In an attempt to discover selective amino acids in G related to pathogenicity of RABV, and consequently provide some new referenced sites to generate highly attenuated rabies vaccines, we compared and analyzed the amino acid sequences of G from different RABV strains and conducted artificial amino acid mutation(s) on the backbone of GD-SH-01, with the corresponding amino acid(s) of HEP-Flury, which is one of the most attenuated rabies fixed strains and does not kill adult mice and was widely used as a vaccine strain (Fox et al., 1957; Sharpless et al., 1957; Morimoto et al., 2011). The pathogenicity of all mutants was then tested in adult mice. Here, we described a new amino acid site of RABV that related to its pathogenicity.

## Materials and Methods

### Cells, Viruses, and Animals

Mouse neuroblastoma (NA) cells (Wuhan Institute of Biological Products, Wuhan, China) were cultured in Roswell Park Memorial Institute (RPMI) 1640 medium (Gibco, Suzhou, China) with 10% fetal bovine serum (FBS) (Gibco, Grand Island, NY, United States). Baby hamster kidney (BHK-21) cells (Wuhan Institute of Biological Products, Wuhan, China) were maintained in Dulbecco’s modified Eagle’s medium (DMEM) (Gibco, Suzhou, China) containing 10% FBS. HEP-Flury (from Jiangsu Academy of Agricultural Sciences, China) were propagated in NA cells. The virulent wild-type strain GD-SH-01 was previously isolated from a rabid pig (Luo et al., 2013). Mutant RABV containing Arg_333_→Gln_333_ of G on the backbone of GD-SH-01 was rescued by our laboratory previously (unpublished data, here as a positive control of attenuated RABV strain). Female Kunming (KM) mice (6–7 weeks old), which are an outbreeding strain from Swiss mice, were purchased from the Center for Laboratory Animal Science of the Southern Medical University (Guangzhou, China). All animal experiments were performed under specific pathogen-free conditions in the Laboratory Animal Center of South China Agricultural University. All animal experiments were approved by the Ethics Committee for Animal Experiments of the South China Agricultural University and conducted in compliance with National Institutes of Health (NIH) guidelines (Zhang et al., 2016). All possible efforts were made to minimize the suffering of laboratory animals.

### Selection of Amino Acid in G Protein

To choose the potential amino acids that could attenuate RABV GD-SH-01 strain (Luo et al., 2012), several pathogenic and non-pathogenic RABV strains were selected, and the amino acid sequences of the G’s ectodomain were compared, using MEGA 6 software [MEGA 6.06 (6140226)]. In this study, we selected partial strains including wild-type strains (from different hosts) and attenuated strains (pathogenic and non-pathogenic strains), together with interested wild-type strain GD-SH-01 (Table 1). The selected viral sequences were obtained from GenBank.

**TABLE 1:** Various rabies virus (RABV) strains information and selected amino acid position.

Strains information				Amino acid position						
Strain	Host	GenBank No.	Passage	G19	G96	G132	G194	G243	G333	G349
HEP-Flury	Human	GU565704.1	+	L	A	L	H	M	Q	E
GD-SH-01	Pig	JX088694.1	wt	I	S	F	N	I	R	G
JX08-48	Ferret badger	FJ719752.1	wt	I	S	F	N	I	R	G
GX4	Dog	DQ849071.1	wt	I	S	F	N	I	R	G
HN10	Human	EU643590.1	wt	I	S	F	N	I	R	G
F04	Ferret badger	FJ712196.1	wt	I	S	F	N	I	R	G
WH11	Donkey	JQ647510.1	wt	I	T	L	N	I	R	G
SH06	Dog	FJ418886.1	wt	I	A	L	N	I	R	G
CYN1009D	Dog	JQ730682.1	wt	I	A	L	N	I	R	G
BJ2011E	Equine	JQ423952.1	wt	I	A	L	N	I	R	G
CQ92	Dog	DQ849072.1	wt	I	A	L	N	I	R	G
RC-HL	Cattle	AB009663.2	+	I	A	L	N	M	R	G
Ni-CE	Cattle	AB128149.1	+	I	A	L	N	M	R	G
CVS-11	Cattle	GQ918139.1	+	I	A	L	N	M	R	G
CVS-B2c	Cattle	AF042824.1	+	I	A	L	N	M	R	G
CVS-N2c	Cattle	HM535790.1	+	I	A	L	N	M	R	G
SAD-B19	Dog	M31046.1	+	I	A	L	N	M	R	G

*“+”, virus has been passaged in non-original host or culture cells; “wt,” wild-type virus.*

Amino acid site that determine pathogenicity of RABV is regularly different between pathogenic strains and non-pathogenic strains. Therefore, we selected amino sites that are different between pathogenic strains and non-pathogenic strains or between wild-type strains and attenuated strains. Therefore, selective amino acids, which differed in selected strains in the G of HEP-Flury were chosen: amino acid at position 19 in G (G19), G194, and G349. In addition, different amino acid residue of G243 between wild-type and attenuated strains was selected. Amino acid residues in the G protein of GD-SH-01 that are different from most other selected strains were chosen: G96 and G132. The selected strains’ information and amino acids are shown in Table 1. All the selected amino acid sites above were mutated artificially on the backbone of GD-SH-01 with the corresponding amino acid of HEP-Flury.

### Construction of the Mutant Full-Length Genome cDNAs and Rescue of the Viruses

The plasmid containing the full-length genome complementary DNA (cDNA) of GD-SH-01 (rGDSH) was constructed and described previously (Tian et al., 2017). To construct the full-length genome cDNA that contains the G19, G96, G132, G194, G243, or G349 mutations of GD-SH-01, the plasmid of rGDSH was amplified using respective primers (Table 2). The amino acids at respective position of GD-SH-01 were replaced with the corresponding amino acids of HEP-Flury. The seamless cloning was performed using One Step Cloning Kit (Vazyme Biotech, Nanjing, China) according to the manufacturer’s protocols. Successful insertion was confirmed by DNA sequencing. All the mutant viruses were rescued in BHK-21 cells as described previously (Inoue et al., 2003; Luo et al., 2017). Rescued viruses were confirmed in NA cells by direct fluorescent antibody assay (dFA) with fluorescein isothiocyanate (FITC)-labeled anti-RABV N antibodies (Fujirabio Diagnostics, Malvern, PA, United States).

**TABLE 2:** Primers used for construction of the mutant full-length genome complementary DNAs (cDNAs).

Primers	Sequences of primers	nt changes
rGDSH-G19-F	CCCATTGAT**T**TACATCATCTCAGCTGTCCGAATAATTTGGTTGTGG	ATA→TTA
rGDSH-G19-R	TGAGATGATGTA**A**ATCAATGGGACTCCAGGGACCGAGTTTGTCTG	
rGDSH-G96-F	TGCGTGCAGA**G**CCGCATACAATTGGAAGATGGCTGGTGACCCCAG	TCC→GCC
rGDSH-G96-R	ATTGTATGCGG**C**TCTGCACGCATCCGGTGTTGGTCGAAAGTGC	
rGDSH-G132-F	AAAGAGTCC**C**T**C**GTCATCATATCTCCAAGTGTGGCAGATCTAG	TTT→CTC
rGDSH-G132-R	TATGATGAC**G**A**G**GGACTCTTTGGTGGTTTTTACAGTCCGGAGC	
rGDSH-G194-F	ATTTTCACC**C**A**T**AGCAGAGGGAAGAGAGCATCCAAAGGGAGC	AAC→CAT
rGDSH-G194-R	CCCTCTGCT**A**T**G**GGTGAAAATATCACAAGAGGTTCCCAGTCTG	
rGDSH-G243-F	TGGGTCGCAAT**G**CAGACATCAGACGAGACCAAGTGGTGCCCTC	ATT→ATG
rGDSH-G243-R	CTGATGTCTG**C**ATTGCGACCCATGTTCCATCCATAAGTCTAAG	
rGDSH-G349-F	AGAGTTGGAG**AG**AGATGTCATCCCCATGTGAACGGGGTGTTTTTC	GGC→GAG
rGDSH-G 349-R	GATGACATCT**CT**CTCCAACTCTCAAACACCCTTTAGAGGGGATG	

*Underlined and bold are nucleotides in the G of GD-SH-01 were replaced by the corresponding nucleotides of HEP-Flury. F, forward primer; R, reverse primer; nt, nucleotides.*

### Virus Propagation and Titration

All the rescued mutant viruses (rGDSH-G19, rGDSH-G96, rGDSH-G132, rGDSH-G194, rGDSH-G243, and rGDSH-G349), rGDSH-G333, HEP-Flury and GD-SH-01 were propagated in NA cells (Neuro-cells which is sensitive to RABV). Virus titers were determined by dFA as described previously (Luo et al., 2016). Briefly, NA or BHK-21 cells grown in 96-well cell-culture plates were inoculated with 10-fold serial dilutions of the indicated virus in RPMI 1640 medium and incubated at 37°C with 5% CO2 for 2 days. Then, culture medium was discarded and cells were fixed with 80% acetone for 30 min at −20°C. Cells were washed with phosphate-buffered saline (PBS) three times and then stained with FITC-labeled anti-RABV N antibodies at 37°C for 60 min. Subsequently, antigen-positive foci were counted under a fluorescence microscope (AMG, Washington, United States), and virus titers were calculated as focus forming units (FFUs) per milliliter (FFU/ml) using the Karber method (Ramakrishnan, 2016).

### Pathogenicity of RABV in Adult Mice

Pathogenicity of mutant strains was conducted in adult mice. KM mice (6–7 weeks of age) were inoculated intramuscularly (i.m.) with 1.0 × 10^5^ FFU or intracerebrally (i.c.) with 2.0 × 10^3^ FFU of HEP-Flury, GD-SH-01, rGDSH-G19, rGDSH-G96, rGDSH-G132, rGDSH-G194, rGDSH-G243, rGDSH-G349, or rGDSH-G333 (as a positive control of attenuated RABV strain). Each group consisted of five or six mice. Mortality was recorded daily for 21 days.

### Virus Growth Curve in NA Cells

Monolayer cultures of 2 × 10^6^ NA cells were infected with virus at a multiplicity of infection (MOI) of 0.1 FFU. Cells were then incubated at 37°C and harvested at 24, 48, 72, and 96 h postinoculation (hpi). Virus titers of samples were determined in NA cells by dFA, as described above. All titrations were carried out in triplicate.

### Virus Spread Assay

The virus spread assay was performed in NA cells in 60-mm cell cultural dishes as described previously (Mei et al., 2019). Briefly, monolayer NA cells were infected with RABV at an MOI of 0.01 and incubated at 37°C. Cells were stained with FITC-labeled anti-RABV N antibodies at 24, 36, 48, and 60 hpi. Fluorescent foci in each dish were observed under a fluorescence microscope. The diameter of each fluorescent foci was measured using Adobe Photoshop CS software (Adobe, San Jose, United States) based on its scale. At least six fluorescent foci in each dish were measured.

### Virus-Neutralizing Antibody Investigation in Adult Mice

Groups of five KM mice (6–7 weeks of age) were inoculated via the i.m. injection of 1.0 × 10^5^ FFU rGDSH-G349 or GD-SH-01. RPMI 1640 medium was used for mock infection. Serum was collected from caudal vein at 9 days postinfection (dpi) and used to determine VNA levels by means of fluorescent antibody virus neutralization (FAVN) tests, as described previously (Cliquet et al., 1998).

### Flow Cytometry

Flow cytometry was carried out to investigate the percentage of immune cells in the spleen after RABV infection. Briefly, KM mice (6–7 weeks of age) were infected i.m. with 1.0 × 10^5^ FFU of rGDSH-G349 or GD-SH-01 or RPMI 1640 medium, respectively. Mouse spleen were harvested at 9 dpi. Single-cell suspensions were prepared followed by treatment with red blood cell lysis buffer (Beyotime, Shanghai, China) following the manufacturer’s instructions, and stained with antibodies against markers of T cells (FITC-CD3e, PE-CD4, PerCP-Cy5.5-CD8a) and B cells (FITC-CD19, PE-CD40) (all antibodies were purchased from Affymetrix eBioscience, United States) by incubation for 30 min on ice. A minimum of 50,000 events were counted using CytoFLEX flow cytometer (Beckman Coulter, United States). Data were analyzed using FlowJo software (Tree Star, Ashland, United States).

### Quantitative Real-Time PCR

Kunming mice (6–7 weeks of age) were inoculated via the i.m. injection of 1.0 × 10^5^ FFU rGDSH-G349 or GD-SH-01. At 5 and 9 dpi, mice were anesthetized with ketamine/xylazine (100/10 mg/kg) and then perfused by intracardiac injection of PBS. Whole brain tissues were harvested and then lysed in Magzol reagent (Magen, Guangzhou, China). Groups of three mice were used for each virus at one time point. Total RNA of each brain tissue sample was extracted using the HiPure Universal RNA Kit (Magen, Guangzhou, China) according to the manufacturer’s protocol. Reverse transcription was carried out using the RevertAid First Strand cDNA Synthesis Kit (Thermo Fisher Scientific, United States) following the manufacturer’s instructions. Quantitative real-time PCR (qRT-PCR) was performed using SYBR Green Master Mix (Vazyme Biotech Co., Ltd., Nanjing, China) in a CFX Connect Real-Time System (Bio-Rad, Hercules, CA, United States). Expression levels of interferon alpha (IFN-α) and immunoglobulin G (IgG) Ê-L chain were normalized to the house-keeping gene glyceraldehyde-3-phosphate dehydrogenase (GAPDH). Genomic RNA was determined with primers amplifying leader RNA and partial N. Primers used to amplify target and reference genes were described previously (Luo et al., 2017).

### Measurement of Viral Load in Periphery

To evaluate RABV load at the inoculation site, KM mice (6–7 weeks of age) were inoculated in the right hind leg with 1.0 × 10^5^FFU rGDSH-G349 or GD-SH-01. After the mice were humanely killed, the right hind leg muscles of five mice from each group were removed from infected mice at 1, 3, 5, 7, and 9 dpi and grinded in liquid nitrogen and then lysed in Magzol reagent (Magen). Total RNA was extracted using the HiPure Universal RNA Kit (Magen), and qRT-PCR was performed as described previously (Wang et al., 2014) to determine RABV genomic RNA in muscles using primers amplifying leader RNA and partial N. Genomic RNA was normalized to GAPDH.

### Virus Growth Curve in Mouse Brain

Rabies virus growth curves *in vivo* were performed in mouse brain. KM mice (6–7 weeks of age) were inoculated i.m. with 1.0 × 10^5^ FFU of rGDSH-D255G or GD-SH-01 in 30 μl RPMI 1640 medium. Three infected mice of each group were euthanized at 1, 3, 5, 7, and 9 dpi, and brains were harvested to detect the RABV genome using qRT-PCR as described previously (Luo et al., 2018). Three infected mouse brains were homogenized in a ninefold volume of RPMI 1640 medium and centrifuged at 12,000 × *g* for 10 min at 4°C following repeated freezing and thawing to investigate virus titer in brains. Supernatants were harvested, and virus titer was determined as described above.

### Measurement of BBB Permeability Using Sodium Fluoride Uptake

Groups of three KM mice (6–7 weeks of age) were inoculated via the i.m. injection of 1.0 × 10^5^ FFU rGDSH-G349 or GD-SH-01. The mock-infected mice were treated with RPMI 1640 medium. Blood–brain barrier (BBB) permeability was measured through the uptake of sodium fluoride as described previously (Luo et al., 2018) at 1, 3, 5, and 9 dpi. Data are expressed as fold change relative to mock-infected mice.

### Statistical Analysis

Data were analyzed using GraphPad Prism 6 software (GraphPad Software, San Jose, CA, United States). The statistical significance was determined using the Student’s *t-*test. *P* < 0.05 was considered to be significantly different.

## Results

### Rescue of Mutant Viruses

**FIGURE 1:**
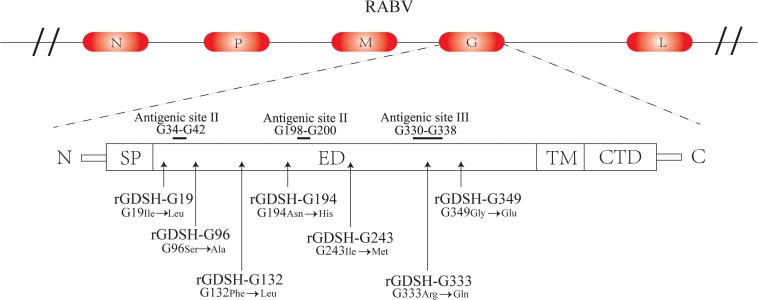
Schematic diagrams of mutations in glycoprotein of rabies virus (RABV). N, nucleoprotein; P, phosphoprotein; M, matrix protein; G, glycoprotein; L, RNA-dependent RNA polymerase. SP, signal peptide; ED, ectodomain; TM, transmembrane domain; CTD, cytoplasmic tail.

Based on the full-length cDNA sequence of GD-SH-01, amino acid (Ile) at G19 was replaced by Leu (Ile_19_→Leu_19_), designated as rGDSH-G19; amino acid (Ser) at G96 was replaced by Ala (Ser_96_→Ala_96_), designated as rGDSH-G96; amino acid (Phe) at G132 was replaced by Leu (Phe_132_→Leu_132_), designated as rGDSH-G132; amino acid (Asn) at G194 was replaced by His (Asn_194_→His_194_), designated as rGDSH-G194; amino acid (Ile) at G243 was replaced by Met (Ile_243_→Met_243_), designated as rGDSH-G243; and amino acid (Gly) at G349 was replaced by Glu (Gly_349_→Glu_349_), designated as rGDSH-G349. Mutant RABV containing Arg_333_→Gln_333_ of G on the backbone of GD-SH-01 was termed rGDSH-G333 (Figure 1). The mutant strains were rescued in BHK-21 cells and each virus was verified in NA cells by immunofluorescence staining using FITC-conjugated antibodies against RABV N protein. Successful single amino acid mutation was confirmed by DNA sequencing.

### Pathogenicity of Mutant Strains in Adult Mice

**FIGURE 2:**
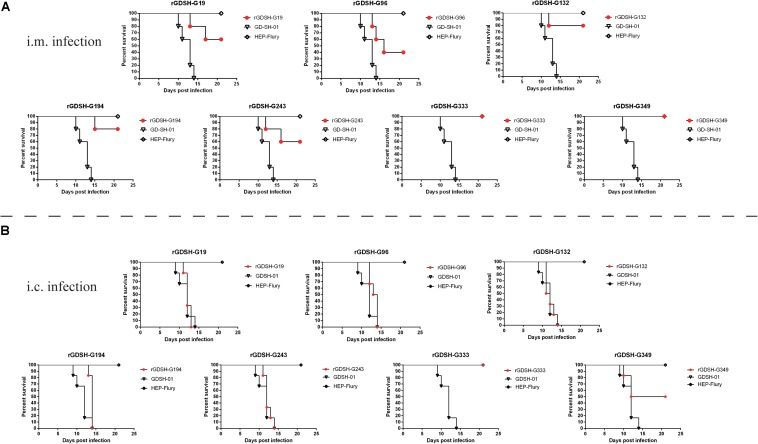
Pathogenicity of mutant rabies virus (RABV) strains in adult mice. Female Kunming (KM) mice (6–7 weeks of age) were inoculated i.m. **(A)** with 1.0 × 10^5^ or i.c. **(B)** with 2.0 × 10^3^FFU of HEP-Flury, GD-SH-01, rGDSH-G19, rGDSH-G96, rGDSH-G132, rGDSH-G194, rGDSH-G243, rGDSH-G333, or rGDSH-G349. Each group consisted of five or six mice. Mortality was recorded daily for 21 days.

To investigate if the selected mutations really attenuated the pathogenicity of RABV, adult KM mice (6–7 weeks) were i.m. or i.c. inoculated with each mutant strain. As shown in Figure 2, virulent strain GD-SH-01 caused 100% mortality by 14 dpi, while, as expected, all the mice survived the infection with the avirulent HEP-Flury through both i.m. and i.c. infection. As shown in Figure 2A, mutants rGDSH-G19, rGDSH-G96, rGDSH-G132, rGDSH-G194, and rGDSH-G243 caused 40, 60, 20, 20, and 40% mortality, respectively, while rGDSH-G349 caused no mice death through i.m. infection, same as the contrast group rGDSH-G333. As shown in Figure 2B, mutants rGDSH-G19, rGDSH-G96, rGDSH-G132, rGDSH-G194, and rGDSH-G243 caused 100% mortality, while rGDSH-G349 caused 50% mortality through i.c. infection. Contrast group rGDSH-G333 did not kill adult mice through i.c. infection (Figure 2B). These results indicated that mutations of G19, G96, G132, G194, or G243 decreased parental pathogenicity after i.m. inoculation, whereas they displayed the same level of pathogenicity as the parent GD-SH-01 after i.c. inoculation. In contrast, the G349 mutation showed to be a promising mutation, as it significantly attenuated GD-SH-01 without killing adult mice through i.m. inoculation.

### Virus Growth Curve in NA Cells

**FIGURE 3:**
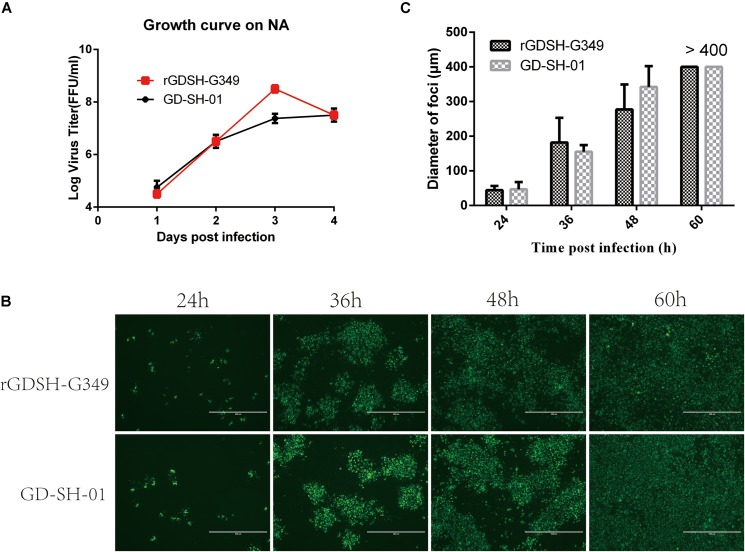
Growth curves and spread ability of rabies virus (RABV) in NA cells. **(A)** Growth curves. NA cells were infected with rGDSH-G349 or GD-SH-01, respectively, at a multiplicity of infection (MOI) of 0.1. At 1, 2, 3, and 4 dpi, culture supernatants were harvested, and virus titers were determined. **(B,C)** Viral spread in NA cells. Monolayer NA cells were infected with rGDSH-G349 or GD-SH-01 at an MOI of 0.01 and incubated at 37°C. Cells were stained with fluorescein isothiocyanate (FITC)-labeled anti-rabies virus (RABV) N antibodies at 24, 36, 48, and 60 hpi. The stained cells were examined under a fluorescence microscope, and for each group, the presentative image out of three replicates was shown **(B)**. **(C)** The diameter of each fluorescent foci was measured based on its scale. Data are presented as mean values ± SE.

Gly_349_→Glu_349_ of G significantly decreased RABV pathogenicity as described above. We therefore will focus on the investigation of rGDSH-G349. The *in vitro* growth curves of rGDSH-G349 were investigated in NA cells. As shown in Figure 3A, G349 mutation strain showed same growth curves compared with parent GD-SH-01. However, rGDSH-G349 reached the highest virus titers at 72 hpi, which was higher than GD-SH-01.

### Spread of Viruses in NA Cells

G exposed on the surface of virion that is responsible for the interaction with host cells. Here, we investigated whether G349 mutations affect viral spread in NA cells. As shown in Figures 3B,C, Gly_349_→Glu_349_ mutation in G did not affect viral spread compared with parent GD-SH-01.

### Immunogenicity of rGDSH-G349 in Adult Mice

**FIGURE 4:**
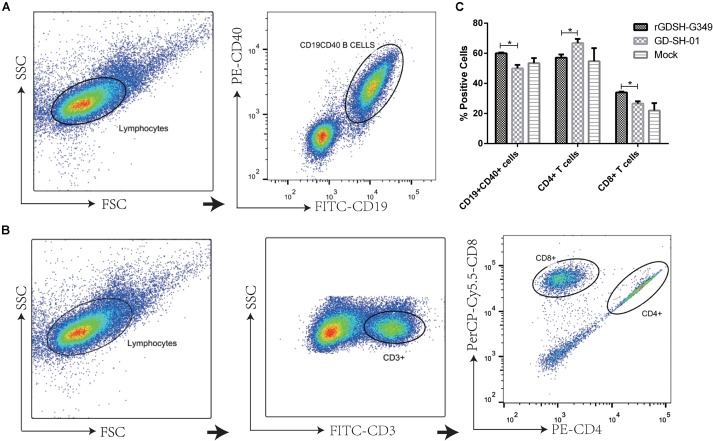
Flow cytometric analysis of immune cells in spleen. Female KM mice (6–7 weeks of age) were infected i.m. with 1.0 × 10^5^FFU of rGDSH-G349, GD-SH-01, or medium alone (mock infection). Spleens were harvested at 9 dpi, and single-cell suspensions were prepared and stained with antibodies against markers of T cells (FITC-CD3e, PE-CD4, PerCP-Cy5.5-CD8a) and B cells (FITC-CD19, PE-CD40). Data were collected and analyzed with a CytoFLEX flow cytometer (Beckman Coulter) and FlowJo software (Tree Star). **(A)** Representative flow cytometric pseudocolor showing the gating strategy to identify CD19 + CD40 + B cells. **(B)** Representative flow cytometric pseudocolor showing the gating strategy to identify CD4 + T cells and CD8 + T cells. **(C)** Percentages of CD19 + CD40 + B cells, CD4 + T cells and CD8 + T cells in spleen (*n* ≥ 3 per group). Values are presented as mean ± SE. Asterisks indicate significant differences between groups, as calculated by Student’s *t*-test (*P < 0.05).

After discovering that rGDSH-G349 significantly attenuated GD-SH-01 pathogenicity, an attempt was made to investigate its immunogenicity, which is essential in the clearance of RABV (Kaplan et al., 1975; Hooper et al., 1998). To investigate the immune response after Gly_349_→Glu_349_ mutation, flow cytometry was conducted to determine contents/counts of CD19 + CD40 + B cells, CD4 + T cells, and CD8 + T cells in spleen at 9 dpi. Figure 4A illustrates the gating strategy to identify CD19 + CD40 + B cells, and Figure 4B illustrates the gating strategy to identify CD4 + T cells and CD8 + T cells. As shown in Figure 4C, rGDSH-G349 recruited more CD19 + CD40 + B cells and CD8 + T cells (from CD3 + T cells) than parent GD-SH-01 in the spleen after i.m. infection. The counts of CD4 + T cells (from CD3 + T cells) induced by GD-SH-01 were more than those induced by rGDSH-G349.

**FIGURE 5:**
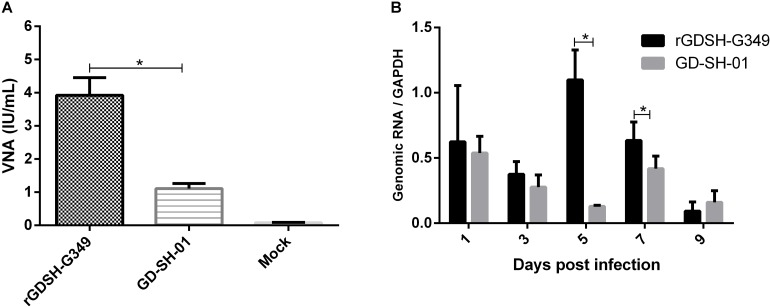
**(A)** Virus neutralizing antibody (VNA) in periphery. Groups of five female Kunming (KM) mice (6–7 weeks of age) were inoculated i.m. with 1.0 × 10^5^ FFU of rGDSH-G349, GD-SH-01 or medium alone (mock infection). Peripheral blood was obtained at 9 dpi, and serum virus-neutralizing antibody was ascertained using fluorescent antibody virus neutralization test, as described in section “Materials and Methods.” **(B)** Viral load at inoculation site. KM mice (6–7 weeks of age) were inoculated in the right hind leg with 1.0 × 10^5^ FFU rGDSH-G349 or GD-SH-01 and the right hind leg muscles were collected from infected mice at 1, 3, 5, 7, and 9 dpi. Genomic RNA of RABV were investigated by quantitative real-time PCR in a CFX Connect Real-Time System. Expression level were normalized to the housekeeping gene GAPDH messenger RNA (mRNA). Data were analyzed using BioRad CFX Manager and GraphPad Prism 6. Results were shown as the mean ± SE. Asterisks indicate significant differences between groups, as analyzed by *t*-test (*P < 0.05).

Virus neutralizing antibody in the periphery blood was determined at 9 dpi in mice after i.m. infection. As shown in Figure 5A, rGDSH-G349 was able to induce a higher level of VNA in periphery compared with parent GD-SH-01. In sum, Gly_349_→Glu_349_ mutation in GD-SH-01 strain enhances its immunogenicity in mice.

### Viral Load of RABV at Inoculation Site

Viral load of RABV at the inoculation site was evaluated in KM mice after i.m. infection with rGDSH-G349 or GD-SH-01. As shown in Figure 5B, more genomic RNA of rGDSH-G349 than parent GD-SH-01 were determined at 5 and 7 dpi. Comparable viral load were detected in mice infected with rGDSH-G349 and GD-SH-01 at 1, 3, and 9 dpi.

### Immune Effectors in CNS After RABV Infection

**FIGURE 6:**
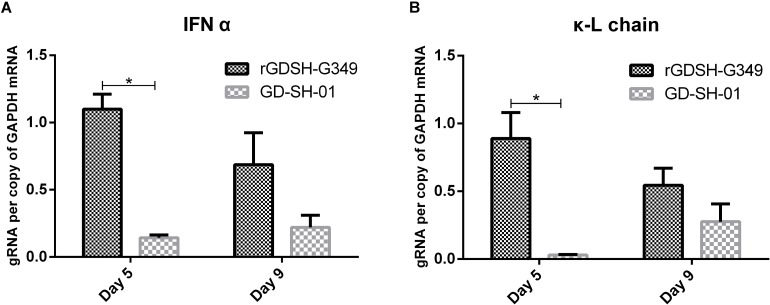
Expression of interferon alpha (IFN-α) **(A)** and immunoglobulin G (IgG) Ê-L chain **(B)** in the CNS. Female adult Kunming (KM) mice were inoculated i.m. with 1.0 × 10^5^ FFU of rGDSH-G349 orGD-SH-01. At 5 and 9 dpi, mice were anesthetized, and brains were harvested following the perfusion with PBS. Expression of interferon alpha (IFN-α) and immunoglobulin G (IgG) Ê-L chain in brain tissues were investigated by quantitative real-time PCR in a CFX Connect Real-Time System. Expression level were presented per copy of the housekeeping gene glyceraldehyde-3-phosphate dehydrogenase (GAPDH) messenger RNA (mRNA0. Results were shown as the mean ± SE. Asterisks indicate significant differences among groups, as calculated by Student’s *t-*test (*P < 0.05).

To investigate the innate immune response caused by RABVs in CNS, mice were immunized with rGDSH-G349 or GD-SH-01 via i.m. route. The messenger RNA (mRNA) levels of IFN-α was investigated using qRT-PCR. As shown in Figure 6A, rGDSH-G349 induced more IFN-α than parent GD-SH-01 at 5 dpi. This indicated that G349 mutation induced a stronger innate immune response in CNS at early stage.

IgG κ-L chain mRNA expression was determined to evaluate antibody level in brain tissues (Phares et al., 2006; Lebrun et al., 2015). As shown in Figure 6B, rGDSH-G349 induced more expression of IgG Ê-L chain than parent GD-SH-01 at 5 dpi. rGDSH-G349 infection triggered more immune effectors infiltrated into CNS than GD-SH-01.

### Virus Growth Curve in Mouse Brain

**FIGURE 7:**
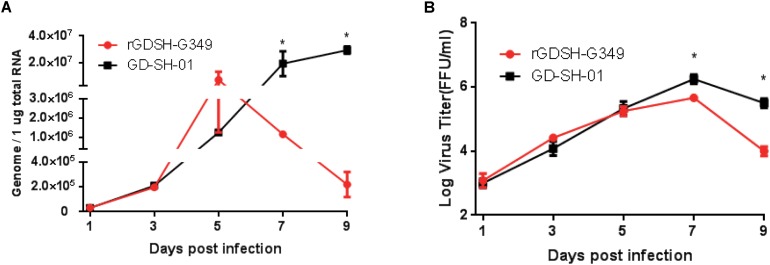
Growth curves of rGDSH-G349 and GD-SH-01 in mouse brain. Kunming (KM) mice were inoculated with 1.0 × 10^5^ FFU of rGDSH-G349 or GD-SH-01 via an i.m. route. Mice were euthanized at 1, 3, 5, 7, and 9 dpi, and brains were harvested to determine rabies virus (RABV) genome **(A)** using quantitative real-time PCR and live virus titers **(B)** as described in *Materials and Methods*. Three mice were used for RABV genome investigations, and three mice were used for virus titers investigations per group. Data are presented as mean values ± SE. Asterisks indicate significant differences between the two groups, as calculated using Student’s *t*-test (*P < 0.05).

Mice were inoculated i.m. with rGDSH-G349 or GD-SH-01, and viral genomes and live virus particles were detected at various times postinoculation to further investigate whether G349 mutation affect virus replication in the CNS. Comparable viral genome and live virus particle levels were detected in mice infected between rGDSH-G349 and GD-SH-01 at 1, 3, and 5 dpi (Figure 7). However, the levels of viral genome and live virus particle of rGDSH-G349 were significantly lower than that of GD-SH-01 at 7 and 9 dpi (Figure 7).

### BBB Permeability Caused by rGDSH-G349 in Adult Mice

**FIGURE 8:**
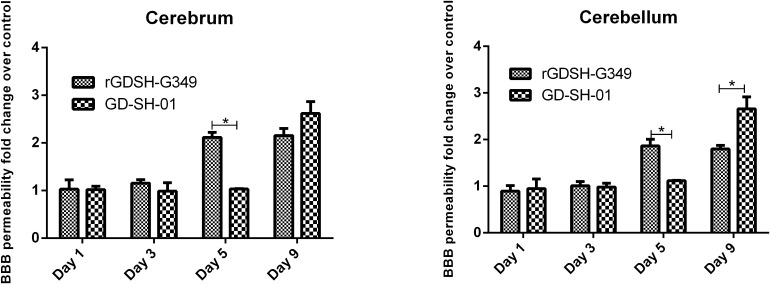
Blood–brain barrier (BBB) permeability in the cerebrum and cerebellum of mice infected with rabies virus (RABV). Kunming (KM) mice (6–7 weeks old) were inoculated i.m. with either rGDSH-G349 or GD-SH-01. RPMI 1640 medium was used for the mock infection. At 1, 3, 5, or 9 dpi, brains were harvested, and BBB permeability was measured by NaF uptake in the cerebrum and cerebellum. Data are presented as fold change over mock infection. *Indicate significant differences among groups, as calculated by Student’s *t-*test (*P < 0.05).

Enhanced BBB permeability may contribute to the clearance of infected RABV in CNS. Here, we investigated whether G349 mutation changed BBB permeability after infection. As shown in Figure 8, mice infected with rGDSH-G349 exhibited increased levels of NaF compared with GD-SH-01 in both the cerebrum and cerebellum at 5 dpi. This suggests that G349 mutation strain enhanced BBB permeability.

## Discussion

Reverse genetic technology is a powerful tool to investigate RABV, and it has been used since the first RABV was rescued from cloned cDNA (Schnell et al., 1994). Since then, several studies have successfully used reverse genetic technology to construct mutant strains of RABV to investigate their pathogenicity or immunogenicity (Takayama-Ito et al., 2006a, b; Yamada et al., 2014b; Nakagawa et al., 2017). Previous studies have confirmed that Ala_242_, Asp_255_, Ile_268_, Lys_330_, and Arg_333_ are all involved in viral pathogenicity (Tuffereau et al., 1989; Coulon et al., 1998; Faber et al., 2005; Takayama-Ito et al., 2006b), suggesting that more than one amino acid are associated with RABV pathogenicity. Here, we speculate that, in addition to the reported amino acid, other novel amino acid sites in G determine the pathogenicity of RABV. By comparing the ectodomain of G from various strains, we selected six potential amino sites, which might be related to pathogenicity of RABV. In this study, mutations were conducted in the backbone of GD-SH-01, which is a highly virulent strain isolated from pig (Luo et al., 2013). To illustrate the relationship between amino acid site and pathogenicity, the viral strain was compared to the avirulent strain HEP-Flury, and the corresponding referenced amino acids were matched and replaced. All the mutant strains were rescued, and the pathogenicity was investigated. Here, we found that Gly_349_→Glu_349_ mutation in G decreased RABV pathogenicity, which was not reported previously.

Interestingly, the comparison of selected G gene sequences highlighted that the HEP-Flury strain exclusively contained Glu at G349, while other strains contained a Gly in that position (Table 1). In addition, only HEP-Flury strain presented Gln at G333, while other strains (in Table 1) contained an Arg. Previous studies had indicated that pathogenicity of RABV decreased significantly when Arg or Lys at G333 was replaced by other amino acids (Seif et al., 1985; Tuffereau et al., 1989; Tao et al., 2010). Therefore, selecting potential sites present in avirulent strains, but not in virulent strains, is an approved strategy for studying the effect of mutation on pathogenicity. Of note, RC-HL strain contains Arg at G333, but it is an avirulent strain (Ito et al., 1994); therefore, mutations related to pathogenicity of RABV are strains dependent. Further studies are necessary to investigate if Glu at G349 could attenuate other RABV strains.

Previous study indicates that by exchanging the ectodomain of G from wild-type with attenuated RABVs, the virus titers and spread ability were altered (Huang et al., 2017). In this study, we discovered that the single mutation at G349 did not significantly alter the virus growth curves and spread ability in NA cells. It has been reported that multiple mutations at positions 252/255/268 of G protein of RC-HL strain did not alter the virus titers in NA cells (Ito et al., 2010). However, single amino mutation at position 37 or 146 of G protein of 1088 strain significantly increased virus replication in NA cells (Yamada et al., 2014b). In addition, mutation of Asn_194_ of G protein attenuated strain SPBNGA with Ser had no effect on virus production, while mutation with Lys decreases virus production (Faber et al., 2005). These previous findings suggest that whether a single or multiple mutations of the G protein of RABV really exerts effect on virus replication is dependent on the specific mutant site or the specific RABV strains, or on both events.

Previous studies indicate that R196S mutation and D247N mutation of G in strain 1088 variants, which led to an additional *N*-glycosylation and decrease in pathogenicity of RABV (Yamada et al., 2014a, b). Therefore, *N*-glycosylation in G may influence pathogenicity of RABV (Yamada et al., 2014b). In this study, G349 mutation does not increase or decrease *N*-glycosylation. Normally, pathogenic RABV infection causes adult mice death while, attenuated RABV does not kill adult mice (Clark, 1978; Morimoto et al., 1998; Takayama-Ito et al., 2004; Shimizu et al., 2007; Tao et al., 2010; Wirblich and Schnell, 2011; Tian et al., 2016; Miao et al., 2017). Here, we found that mutation from Gly to Glu at G349 significantly attenuated GD-SH-01, the same as mutation of G333, without killing adult mice by i.m. inoculation. Neuroinvasiveness do not contribute to the attenuated virulence of G349 mutation because rGDSH-G349 showed the same growth ability compared with parent GD-SH-01 at an early stage of infection in the CNS. There must be other factors that direct the clearance of infected rGDSH-G349. Both innate immune response and adaptive immune response are essential in the clearance of RABV (Kaplan et al., 1975; Hooper et al., 1998; Chopy et al., 2011).

G plays the most important role in the induction of VNA (Cox et al., 1977). A previous study has confirmed that variant 1088-N30 induces higher level of VNA than street strain 1088 in serum in the earlier phase of infection after i.m. inoculation (Yamada et al., 2012). In this study, we found that VNA titer in mice induced by rGDSH-G349 was higher than parent GD-SH-01 after i.m. infection. What’s more, rGDSH-G349 recruited more CD19 + CD40 + B cells and CD8 + T cells in spleen. The immunogenicity of rGDSH-G349 is stronger than GD-SH-01, and this may be due to more viral load at inoculation site as the results showed. In addition, Gly_349_→Glu_349_ mutation attenuated RABV pathogenicity, and this may contribute to the activation of dendritic cells (Yang et al., 2015). In addition, G349 mutation enhanced IgG Ê-L chain and IFN-α expression in CNS. Therefore, the enhanced innate immune response in CNS and infiltration of immune effectors induced by G349 mutation might contribute to the clearance of infected RABV. This explained why the virus load of rGDSH-G349 decreased at a late stage of infection in CNS.

Recent studies have confirmed the crystal structures of RABV G and its interaction with neutralizing antibodies (Hellert et al., 2020; Yang et al., 2020). Residues from G333–G350 of ectodomain from three strains (CVS-11, Flury, and SAD-B19) are observed to bond to antibody 523-11 (Yang et al., 2020). They also found that antibody 523-11 could block the G-mediated syncytia formation of CVS-11, Flury, and SAD-B19 strains. However, antibody 523-11 only inhabits the infection of cells by Flury. The different residues from G333 to G350 were G333 (Gln: Arg) and G349 (Glu: Gly) between Fulry and CVS-11or SAD-B19. In this study, interested G349 locates the region that is a target of neutralizing antibodies, and its mutation may change the interaction with host cells. Further work is needed to confirm this speculation.

Enhanced BBB permeability caused by attenuated RABV also contribute to the clearance of infected RABV (Phares et al., 2006; Roy et al., 2007; Hooper et al., 2009; Chai et al., 2015). In this study, we found that rGDSH-G349 rather than parent GD-SH-01 significantly enhanced BBB permeability at 5 dpi. This might contribute to the enhanced inflammation in CNS and subsequently clear the infected RABV. In addition, VNA in serum may also contribute to the clearance of RABV in the CNS when BBB permeability opens (Huang et al., 2014). rGDSH-G349 induced higher levels of VNA in periphery after i.m. infection.

Previous studies indicated that attenuation caused by a single mutation in G of RBAV may revert according to reverse mutation or new mutation occurred in other amino acid site (Faber et al., 2005; Tao et al., 2010). Multiple amino acid changes could extensively attenuate the pathogenicity of RBAV and improve the stability of attenuation phenotype (Mebatsion, 2001; Dietzschold et al., 2004; Faber et al., 2005; Masatani et al., 2011; Nakagawa et al., 2017). Therefore, G349 could be a potential site when construct multiple amino acid changes to extensively attenuate the RABV pathogenicity.

In summary, by comparing the amino acid sequence of G from different RABV strains, we selected six potential pathogenic amino acid positions. Gly_349_→Glu_349_ mutation in G significantly decreased RABV pathogenicity through enhanced immune response rather than decreased replication. G349 mutation enhanced BBB permeability and might contribute to the clearance of RABV in CNS. In conclusion, this study discovered a new amino acid site of RABV related to pathogenicity.

## Data Availability Statement

The datasets generated for this study are available on request to the corresponding author.

## Ethics Statement

The animal study was reviewed and approved by the Ethics Committee for Animal Experiments of the South China Agricultural University.

## Author Contributions

XG and JL conceived and designed the experiments. JL, BZ, and YW performed the experiments. JL and XG analyzed the data. JL and XG wrote the manuscript. All authors read and approved the final manuscript.

## Conflict of Interest

The authors declare that the research was conducted in the absence of any commercial or financial relationships that could be construed as a potential conflict of interest.
